# Clinical Significance of Time‐to‐Surgery and COVID‐19 Pandemic in Surgically Treated Non‐Small Cell Lung Cancer

**DOI:** 10.1111/1759-7714.70163

**Published:** 2025-09-11

**Authors:** Áron Ghimessy, János Fillinger, Márton Csaba, Sára Lality, Gábor Tarsoly, Hanna Tihanyi, Kristóf Csende, Péter Radeczky, Balázs Gieszer, Levente Bogyó, Klára Török, László Mészáros, Áron Gellért, Bence Ferencz, Balázs Döme, Ákos Kocsis, László Agócs, Ferenc Rényi‐Vámos, Zsolt Megyesfalvi

**Affiliations:** ^1^ Department of Thoracic Surgery National Institute of Oncology Budapest Hungary; ^2^ Department of Thoracic Surgery Semmelweis University Budapest Hungary; ^3^ National Korányi Institute of Pulmonology Budapest Hungary; ^4^ Department of Thoracic Surgery, Comprehensive Cancer Center Medical University of Vienna Vienna Austria; ^5^ Department of Translational Medicine Lund University Lund Sweden; ^6^ National Institute of Oncology and National Tumor Biology Laboratory Budapest Hungary

**Keywords:** COVID‐19 pandemic, NSCLC, time‐to‐surgery

## Abstract

**Objectives:**

Timely discovery and adequate patient management are crucial in non‐small cell lung cancer (NSCLC) since long‐term survival is only achievable in early‐stage disease. In our study, we aimed to elucidate the effects of time to surgery on survival and to assess the impact of the COVID‐19 pandemic on elapsed time until surgery.

**Methods:**

In total, 2536 Caucasian NSCLC patients who underwent curative‐intent lung resection surgery were included in this study. 1 month, 2 months, 77 days, and 91.06 days between CT‐based diagnosis and surgery were evaluated as possible cut‐off values for worse outcome. Survival curves were estimated by Kaplan–Meier plots, and the differences between groups were compared using the log‐rank test. Multivariate analysis was performed using a Cox regression model.

**Results:**

Patients with time‐to‐surgery ≥ 2 months had significantly impaired overall survival (OS) (vs. those with < 2 months; *p* = 0.002). In our multivariate model, time‐to‐surgery (*p* = 0.011), age (*p* = 0.02), diabetes mellitus (*p* = 0.02), disease stage (*p* = 0.0001) and vascular invasion (*p* < 0.001) all had a significant impact on OS. Importantly, during the COVID‐19 pandemic, the elapsed time between diagnosis and surgery increased with a median of 12 days, resulting in a significant delay in time‐to‐surgery compared to the pre‐pandemic period (*p* < 0.001). Post hoc tests showed, however, that there were no significant differences in time‐to‐surgery concerning the major waves of COVID‐19 infections.

**Conclusions:**

Time‐to‐surgery is an independent predictor of long‐term survival in surgically treated NSCLC. In general, the COVID‐19 pandemic caused a significant delay in the elapsed time until surgery, but the specific COVID‐19 waves had no significant impact on time‐to‐surgery.

## Graphical abstract

Delayed surgical treatment with time‐to‐surgery ≥ 2 months had significantly impaired overall survival. During the COVID‐19 pandemic, the elapsed time between diagnosis and surgery increased with a median of 12 days. Time‐to‐surgery is an independent predictor of long‐term survival in surgically‐treated NSCLC, thus oncological services must be protected during a global pandemic.

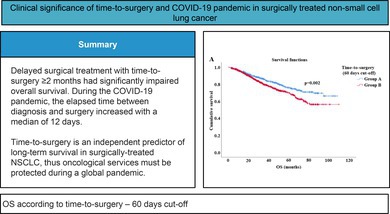

**Abbreviations**
COPD: Chronic Obstructive Pulmonary Disease
COVID‐19: Coronavirus disease 2019
CT: Computer Tomography
DM: Diabetes Mellitus
GERD: Gastro‐Esophageal Reflux Disease
NSCLC: Non‐Small Cell Lung Cancer
OS: Overall Survival
PET: Positron Emission Tomography

## 1 Introduction

Lung cancer is one of the most frequently diagnosed cancers worldwide and contributes to more cancer‐related deaths than any other malignancy [1]. Although the introduction of immune‐checkpoint inhibitors and targeted agents into the armamentarium of lung cancer treatment has markedly improved the survival rates, lung cancer remains an incurable disease for most patients [1, 2, 3, 4]. This is mainly because the vast majority of patients are still diagnosed with advanced‐stage disease when the survival outcomes are considerably worse than in earlier stages [5]. For patients with early‐stage disease, however, surgical resection of the lung lesion represents an attractive treatment option that often offers favorable long‐term outcomes [5].

While recent studies suggest that the age‐standardized incidence and mortality rates of lung cancer in Hungary are lower than previously expected, lung cancer still represents a great socio‐economic burden and its incidence remains high both in men and women [6, 7]. Hence, in order to facilitate early diagnosis and reduce lung cancer‐related mortality, the implementation of screening programs and optimized management protocols is essential. Indeed, the results of the HUNCHEST screening program suggest that volume‐based low‐dose CT screening may facilitate diagnosis, yet the particular aspects of clinical pathways after diagnosis and their impact on survival are still mostly unknown [8].

Although the biological behavior of lung cancer is highly variable, and the rate of tumor growth and metastatic spread is difficult to predict, it is widely accepted that timely treatment is of utmost importance. Several studies have highlighted so far that delayed diagnosis and treatment are directly associated with higher disease stages [9, 10]. Importantly, Serna‐Gallegos et al. reported that even 1 week of delay in treatment can cause upstaging in 21.7% of patients from stage 1 to stage 2 [11]. The work‐up before lung cancer surgery involves several imaging procedures and invasive examinations coordinated by multidisciplinary teams comprising several specialties. There are multiple factors that contribute to delayed surgical resection; however, the effects of this delay on short‐ and long‐term outcomes are rarely studied. Moreover, there is no widely accepted benchmark for time‐to‐surgery. 2 months is regarded as optimal in several centers, but there is data supporting longer waiting times [12, 13]. Mayne et al. found that Stage IA1 NSCLC patients did not have significantly worse survival when surgery was delayed up to 120 days; however, 1A2 to IB patients experienced worse survival [14]. In the last years, the COVID‐19 pandemic put an enormous strain on healthcare systems all over the world, which possibly had a considerable impact on the length of patient work‐up before surgery as well.

In this large‐scale, single‐center retrospective study, we investigated the effects of extended time to surgery on survival and assessed the impact of the COVID‐19 pandemic on the elapsed time between diagnosis and lung resection surgery in non‐small cell lung cancer (NSCLC).

## 2 Patients and Methods

### 2.1 Patients and Inclusion Criteria

The data of 2536 patients who underwent curative‐intent lung resection surgery for NSCLC in the National Institute of Oncology, Budapest, Hungary between 2013 and 2021 was retrospectively analyzed. Clinicopathological and survival data were collected from the medical records and the records of the Central Statistical Office in accordance with the relevant guidelines. Ethical authorization for data analysis was obtained from the local ethical committee, and the need for signed consent was waived due to the retrospective nature of this study. Further details of the study population and treatments are provided in Supporting Information and methods.

### 2.2 Data Collection

The time of lung cancer diagnosis refers to the date of first radiological suspicion as defined by the guidelines of the Fleischner Society: first X‐ray, computer tomography (CT) scan or positron emission tomography (PET) that mentions a pulmonary nodule that requires further examination [15].

**FIGURE 1:**
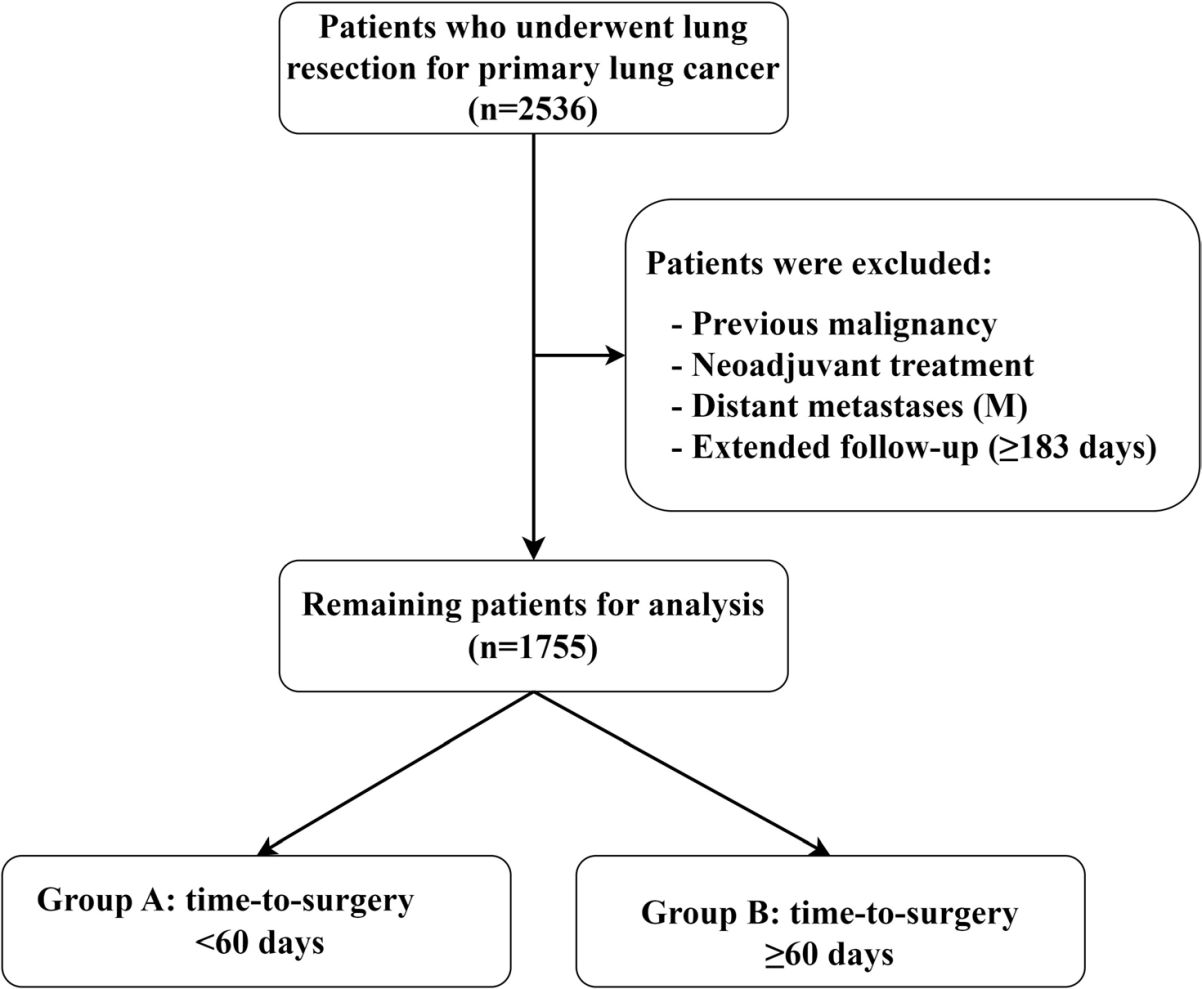
Consort diagram of patient selection. NSCLC, Non‐small Cell Lung Cancer.

Preoperative workup including CT scans, lung function tests, flexible bronchoscopy, and additional staging examinations by abdominal ultrasound, bone scintigraphy, or PET‐CT imaging was generally performed in the corresponding pulmonary centers. Accordingly, two patient subgroups were defined for further analyses. Group A consisted of patients who underwent lung resection within 60 days of first suspicion, whereas patients where the time to surgery was ≥ 60 days were categorized as Group B individuals. The inclusion and exclusion criteria along with the patient selection workflow are summarized in Figure 1.

For further details of the data collection see Supporting Information and methods. The inclusion and exclusion criteria along with the patient selection workflow are summarized in Figure 1.

### 2.3 Survival Analysis

OS was analyzed in function of the clinicopathological characteristics (such as age, diabetes mellitus (DM), chronic obstructive pulmonary disease (COPD), high blood pressure, cardiovascular disease, gastric reflux, adjuvant chemotherapy, tumor stage, vascular invasion) and the elapsed time until surgery. See Supporting Information and methods for further details.

### 2.4 Statistical Analysis

All statistical analyses were performed by using the SPSS Statistics 26.0.1 package (IBM SPSS Statistics, IBM Corp., Armonk, NY, USA). See Supporting Information and methods for details.

## 3 Results

### 3.1 Patient Characteristics

After applying the exclusion criteria, 1755 surgically treated NSCLC patients were enrolled in the study whose clinicopathological characteristics are summarized in Table 1. Group A consisted of 657 individuals, whereas 1098 patients were included in Group B.

**TABLE 1:** Patient characteristics and pathological findings in Group A and B.

Clinicopathological variables	Group A	Group B	*p*
Age (years), median (range)	62 (28–88)	64 (32–85)	0.011
Gender			
Female	372 (58.03%)	668 (59.96%)	0.428
Male	269 (41.97%)	446 (40.04%)	
Histology (%)			
Adenocarcinoma	372 (58.03%)	687 (61.67%)	0.285
Squamous‐cell carcinoma	189 (29.49%)	282 (25.31%)	
Large cell carcinoma	3 (0.47%)	4 (0.36%)	
NSCLC NOS	77 (12.01%)	141 (12.66%)	
Stage (%)			
IA	237 (36.41%)	367 (33.24%)	0.061
IB	136 (20.89%)	219 (19.84%)	
IIA	51 (7.83%)	75 (6.79%)	
IIB	80 (12.29%)	201 (18.21%)	
IIIA	91 (13.98%)	154 (13.95%)	
IIIB	26 (3.99%)	29 (2.63%)	
IV	6 (0.92%)	9 (0.82%)	
N/A	24 (3.69%)	50 (4.53%)	
Grade			
1	348 (54.29%)	538 (48.29%)	0.146
2	118 (18.41%)	223 (20.03%)	
3	112 (17.47%)	154 (13.82%)	
N/A	63 (9.83%)	199 (17.86%)	

Abbreviations: N/A, Not available; NOS, Not otherwise specified; NSCLC, Non‐small cell lung cancer.

Advanced disease stage (*p* < 0.001), vascular invasion (*p* < 0.001), and the presence of DM (*p* < 0.001) were all strong negative prognosticators in the entire cohort. Meanwhile, the presence of COPD (*p* = 0.12), cardiovascular disease (*p* = 0.56), high blood pressure (*p* = 0.65) and GERD (*p* = 0.189) had no significant effect on the OS.

The patient characteristics did not differ significantly between the two groups, although patients were older in Group B than in Group A (age: *p* = 0.011; gender: *p* = 0.428; disease stage: *p* = 0.061; tumor grade: *p* = 0.146, presence of any comorbidities: *p* = 0.34).

Age, the presence of DM, disease stage, vascular invasion, visceral pleural invasion as well as time‐to‐surgery were found to be independent negative prognostic factors with Cox regression analysis (Table 2).

**TABLE 2:** Cox regression analysis for multivariate survival analysis. Age, Stage, Vascular invasion, DM, and the time‐to‐surgery were all independent prognostic factors.

Variables	HR (CI)	*p*
Age	1.028 (1.013–1.043)	< 0.001
Diabetes mellitus	1.459 (1.107–1.925)	0.007
Time‐to‐surgery	1.003 (1.000–1.006)	0.046
Stage	1.605 (1.405–1.834)	< 0.001
Vascular invasion	1.844 (1.446–2.351)	< 0.001
Visceral pleural invasion	1.100 (1.05–1.12)	0.03

Abbreviations: CI, Confidence interval; HR, Hazard ratio.

### 3.2 Survival Analysis

Mean and median time‐to‐surgery was 77 and 91.06 days, respectively, for the entire cohort. The median survival for group A was 86.181 (95% CI: 82755–89 607) months, while for group B it was 79.748 (95% CI: 76403–83 094) months.

**FIGURE 2:**
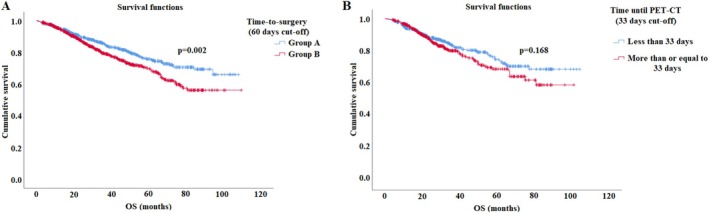
ANOVA analysis revealed increasing temporal trends in average time to surgery increases from 2013 to 2021. ANOVA, Analysis of Variance.

As our study investigates a rather long timespan, we examined whether time‐to‐surgery has changed over time. To rule out this altering effect, we also examined the distribution of operation date between the two groups. As shown in Figure 2, we found that time‐to‐surgery gradually increased from 2013 to 2021. With ANOVA test, we found no significant differences between group A and B regarding operation date distribution (*p* < 0.001).

### 3.3 Investigating the Reasons Behind Delayed Surgical Resection

**FIGURE 3:**
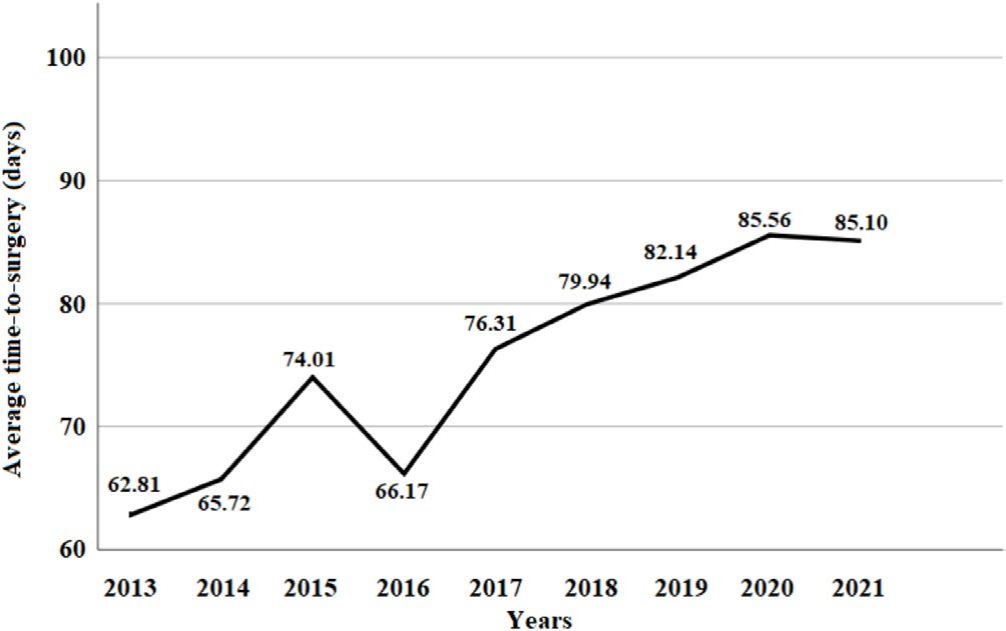
(A) OS according to time‐to‐surgery using 60 days cut‐off. Patients in Group B had significantly shorter survival when compared to patients in Group A (*p* = 0.002). (B) OS of patients according to the delay caused by PET CT imaging in the preoperative work‐up. Better survival was seen in patients where the delay was less than 33 days, although the difference was not statistically significant (*p* = 0.168). OS, Overall Survival; PET‐CT, Positron Emission Tomography – Computed Tomography.

The fact whether PET‐CT was done during the medical work‐up surprisingly had no effect on survival in itself (*p* = 0.724). However, when examining the delay in surgery caused by the wait for PET‐CT, we found a tendency toward improved survival in patients who had a shorter delay (*p* = 0.168; Figure 3B). The mean waiting time for PET‐CT was 33 days, and we used this value as a cut‐off.

**FIGURE 4:**
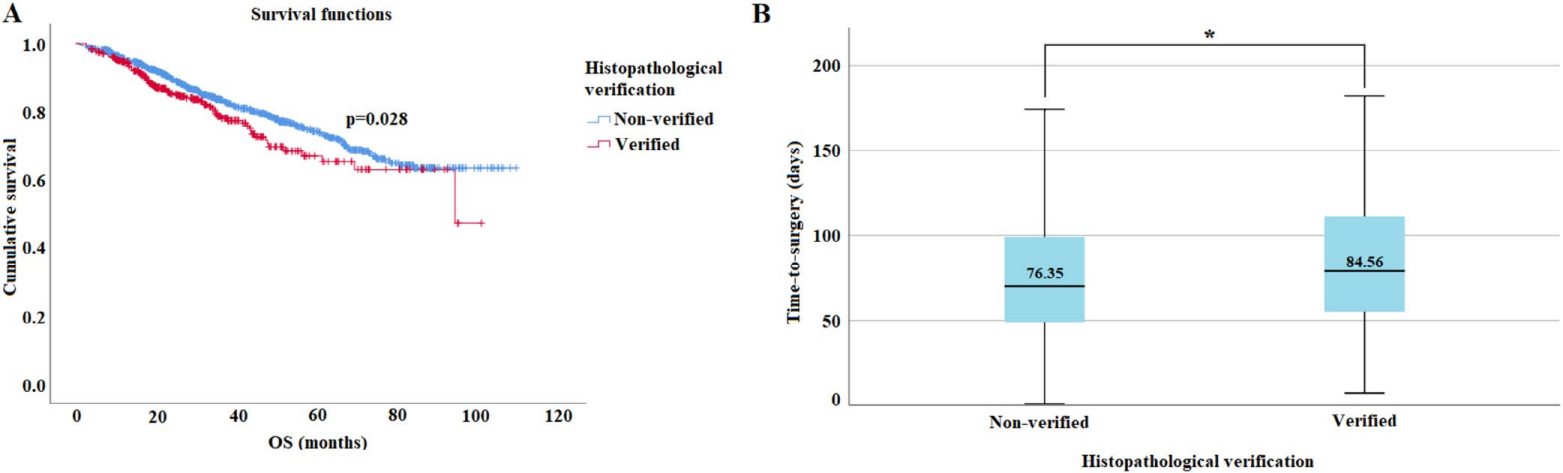
(A) OS stratified by the presence or absence of preoperative tissue verification Patients without tissue verification had better survival (*p* = 0.028). (B) Average delay in surgical work‐up caused by tissue verification, showing a mean of 10 days additional waiting time. OS, Overall Survival.

Interestingly, the fact that a patient underwent preoperative tissue verification was identified as a negative prognostic factor in our cohort (*p* = 0.028). Although there might be multiple contributing factors to this, it is noteworthy that perioperative tissue verification caused a mean of 10‐day delay and even more in patients when the first biopsy was unsuccessful (Figure 4).

No significant difference was seen in the length of time to surgery when comparing the rank of the examining pulmonary centers (national centers vs. local pulmonary centers (outside Budapest) vs. centers in Budapest, *p* = 0.947).

### 3.4 The COVID‐19 Pandemic Significantly Alters the Time‐to‐Surgery in Surgically Treatable NSCLC Patients

**FIGURE 5:**
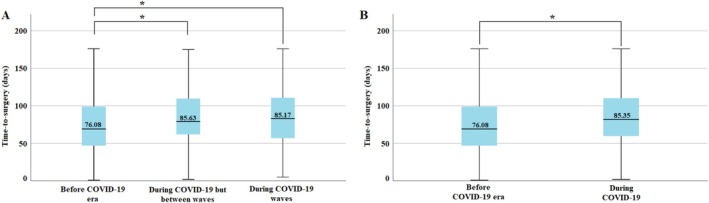
Time‐to‐surgery (work‐up before surgery): (A) comparison of 0—before COVID, 1 during COVID but between waves and 2—during COVID waves. (B) comparison of 0—before COVID, 1 during COVID: Work‐up before surgery became 12 days longer in average since the COVID pandemic. COVID‐19, Coronavirus Disease 2019.

Most notably, COVID‐19 had a negative effect on the elapsed time between the first suspicion of lung cancer and surgery. When comparing the three patient subgroups as defined by the COVID‐19 periods, a significantly longer time‐to‐surgery was seen in patients treated after the outbreak of the pandemic (*p* < 0.001). Specifically, the patient work‐up was on average 12 days longer during COVID‐19 waves and on average 10 days longer between COVID‐19 waves when compared to the time before the COVID‐19 outbreak. Similar differences in work‐up duration were seen when we compared the whole COVID‐19 era to the time before (Figure 5).

Since time‐to‐surgery has been constantly rising since 2013, we performed a subsequent analysis comparing the last 2 years before the pandemic with the first 2 years of the COVID‐19 era to exclude the distorting effect of the constant long‐term rise seen in the pre‐pandemic period. Notably, time‐to‐surgery was statistically significantly higher in the 2020–2021 timeframe than in 2018–2019 (mean: 82 days vs. 75 days, respectively; *p* = 0.048). Average tumor diameter increased from 30.53 mm before the pandemic to 32.52 mm in the cases operated during the COVID‐19 pandemic; however, this difference was not statistically significant (*p* = 0.465). The COVID‐19 pandemic had no statistically significant effect on the stage distribution of our surgically treated patients (*p* = 0.558).

Contrary to this, COVID‐19 had a negative effect on our cohort: before the pandemic, 68.42% of our patients underwent VATS operation for their primary lung cancer; however, after the COVID‐19 outbreak, this value decreased to 59%. Since our selection criteria for minimal‐invasive surgery did not change during this time, the presence of a growth in tumor size or the presence of other contributing factors such as lymph node enlargement are obvious suspicions. We did not detect statistically significant tumor growth or stage shifting, and thus we hypothesize that the effects of undetected or previous COVID infection (such as borderline enlarged lymph nodes or inflammatory consolidations caused us to select an open procedure more often).

## 4 Discussion

Several studies reported that even moderate delays in the treatment of aggressive cancers could have a significant negative effect on survival [16, 17]. The COVID‐19 pandemic disrupted medical care in many ways. Elective surgical procedures were put on hold amid safety concerns, and the number of screening and staging examinations diminished. Therefore, our study primarily aimed to investigate the effects of delayed time to surgery on survival and to evaluate the impact of the COVID‐19 pandemic on the elapsed time between diagnosis and surgery in NSCLC patients.

The doubling time of NSCLC is highly variable; different publications report values between 4 and 56 weeks [18, 19, 20], while the mean doubling time was found to be around 16 weeks. Patients with tumors doubling in less than 16 weeks had significantly worse survival [13]. The majority of our patients who underwent curative‐intent anatomical lung resection surgery had a longer work‐up than 60 days (Group B). A possible explanation behind this longer work‐up time in Group B might lie behind older age; however, the presence of comorbidities was not higher, and usually additional tests were warranted due to comorbidities, not age alone. In this study, a 60‐day cut‐off was found to be of clinical significance in regard to survival. Ponholzer et al. used the same cut‐off value [12], but some centers suggested a longer period up to 12 weeks with an acceptable survival outcome [21]. Some patients in our cohort had considerably longer time to surgery than 12 weeks, and this time seems to worsen long‐term survival. Despite our expectations, no statistically significant upstaging (based on pathological findings) could be seen in patients who had a longer work‐up than 60 days. Importantly, centers that used a longer cut‐off value experienced significant upstaging [21]. One factor that might contribute to worse survival, even when pathological upstaging is not proven, is therapy resistance. It was reported that delay of radiation therapy [22] and chemotherapy [23] can cause therapy resistance and a higher chance of recurrence.

PET‐CT became an integral part of NSCLC patients' staging before surgery [24]. However, according to our findings, if these investigations are delayed due to their (in)accessibility then the negative effect of this delay on survival might outweigh the benefit of a PET‐CT itself. Indeed, Mohammed et al. found that almost 50% of patients have tumor progression on PET‐CT after 8 weeks, suggesting that even a 4‐week delay due to PET‐CT waiting is unacceptable [25]. Healthcare management and pulmonary centers have to address this issue in order to provide better care for NSCLC patients. In the last decade, the waiting times have improved significantly in Hungary. At the beginning of our study period, the mean waiting time was 33 days for the whole cohort but decreased to 20 days when we assessed the patients operated on in the last 2 years. Of note, PET‐CT imaging, one of the most reliable forms of staging methods, has become mandatory for all patients only in recent years. In some instances, this resulted in inadequate preoperative staging, thus increasing the number of stage IIIA and stage IIIB cases. Another reason behind the relatively increased number of IIIA/IIIB cases is that single‐station N2 disease is operated upfront in Hungary.

Our data suggest that preoperative pathological assessment of the lung lesion should be only considered if it causes no or minimal delay in time to surgery or in selected patients. In our cohort, patients with verified tumors had inferior survival outcomes, yet this can also be attributed to selection bias, the fact that larger and centrally located intrabronchial tumors are more likely to be biopsied, which reflects the more aggressive nature of the disease rather than the effect of delay. Our current analysis did not go into detail about the individual effects of each diagnostic step, but future prospective studies with refined clinical data may help to clarify these factors. To date, most protocols recommend endoscopic needle aspiration for nodal staging [24]; however, this can also lead to delays in patient work‐up. Therefore, it is recommended to organize patient work‐up in big volume centers where nodal staging and tissue verification of the tumor can be done without causing significant delay in treatment. The average tumor diameter at surgery was concerningly high before and after the COVID‐19 outbreak. This is mainly because there was no established nationwide low‐dose CT screening program in the country for most of the study period. Nevertheless, a population‐based screening program was recently implemented in Hungary, and interim analysis suggests a shift toward early detection [8].

Lastly, our study reveals that the COVID‐19 pandemic had a significant impact on the time to surgery, causing significant delays in several patients. Interestingly, this delay was also seen between the pandemic waves, suggesting a general strain on the pulmonary network of Hungary, rather than the temporary overwhelming of the system. COVID infection after lung cancer surgery might be a negative prognostic factor in itself. Villena‐Vargas et al. reported a 40% mortality when the COVID infection occurred within 90 days of lung cancer surgery, which suggests that COVID‐19 infection could have a major role on survival during the pandemic [26]. Other reports suggested similar effects, although the time of infection and the extent of resection had a major role [27]. On the other hand, there are several reports of successful surgical treatment after prolonged or persistent COVID infection [28, 29].

In our data, an increase in tumor size and a subsequent decrease in VATS/open ratio was seen; however, we did not see significant upstaging in our patients, contrary to other reports [30, 31, 32, 33]. Although the delay experienced in our center was moderate compared to those reported by others [16], it still had a significant impact on survival. Several indirect indicators also suggest that the pandemic had a negative impact on oncology care. A decrease in the number of minimally invasive surgeries despite unchanged selection criteria, a slight increase in tumor size, and a 12‐day delay in surgeries all suggest a delay in the diagnostic process. Although we did not have data on recurrence‐free survival (RFS) or stage progression, these trends may reflect subclinical tumor progression and highlight the need for more comprehensive outcome measures in future studies; nevertheless, oncological work‐up and treatments of cancer patients have to be maintained as well as possible during pandemics; otherwise, many lives and quality days will be lost as collateral damage.

### 4.1 Limitations

This was a single‐center retrospective study with well‐known limitations. A randomized and prospective study was not feasible on this topic. The cut‐off values we used for time‐to‐surgery were arbitrary and need further validation; thus, they can be used primarily on our own data. Although comorbidities did not differ significantly between patients undergoing early and late surgery, it should be noted that unmeasured factors—such as frailty, functional status, or socioeconomic circumstances—may have influenced both the timing of surgery and the outcomes. These factors were not included in our database and may represent residual confounders. It is important to emphasize that future prospective studies should include standardized assessment of frailty and social determinants of health to better control for selection bias.

A further limitation is that we do not have recurrence‐free survival data, which would be a better indicator of timely surgical resection.

Finally, our study was conducted at a single, high‐volume thoracic surgery center in Hungary, which may limit the generalizability of the results. Although this environment provided uniform diagnostic protocols and consistent surgical care, differences in healthcare system structure, access to diagnostic imaging, and surgical capacity may influence the time to treatment initiation and outcomes in other regions. We now encourage future multicenter or international studies to validate our results in different clinical settings.

## 5 Conclusion

In this retrospective, single‐center study, we report that the elapsed time between the first suspicion of lung cancer and surgery greatly impacts the survival outcomes of NSCLC patients. Specifically, we show that increased time to surgery (> 60 days) is an independent negative prognosticator in these patients. Patient adherence, additional tests due to comorbidities, and the long waiting time for PET‐CT imaging and preoperative tissue verification all contribute to longer time to surgery. Our study is among the first to investigate the impact of the COVID‐19 pandemic on the clinical pathway of surgically treated NSCLC patients. Importantly, time to surgery was significantly shorter in the pre‐pandemic period. Notably, however, the elapsed time until surgery did not differ significantly in the COVID‐19 era between the pandemic waves. This is suggestive of the general strain put on the Hungarian healthcare system. Nationwide healthcare programs are warranted to address the potential delays in the treatment of cancer patients caused by a pandemic.

## Author Contributions

Áron Ghimessy acted as principal investigator and wrote the manuscript. János Fillinger conducted the pathological re‐assessment of the tumor samples. Márton Csaba, Sára Lality, Gábor Tarsoly, Hanna Tihanyi and Kristóf Csende contributed to data collection Péter Radeczky, Balázs Gieszer, Levente Bogyó, Klára Török, László Mészáros have contributed to study design. Áron Gellért and Bence Ferencz were responsible for statistical analysis. Zsolt Megyesfalvi, Balázs Döme, János Fillinger, Ákos Kocsis, László Agócs and Ferenc Rényi‐Vámos were supervisors of the study design, statistical analysis and manuscript.

## Ethics Statement

The study was conducted in accordance with the guidelines of the Helsinki Declaration of the World Medical Association. Ethical authorization for data analysis was obtained from the local ethical committee and the need for signed consent was waived due to the retrospective nature of this study.

## Conflicts of Interest

The authors declare no conflicts of interest.

## Supporting information


**Data S1:** Supporting Information.

## Data Availability

The data underlying this article will be shared on reasonable request to the corresponding author.
