# Supplementary material for: Clinical Significance of Time‐to‐Surgery and COVID‐19 Pandemic in Surgically Treated Non‐Small Cell Lung Cancer
Source: Thorac Cancer. 2025 Sep 11;16(17):e70163. doi: 10.1111/1759-7714.70163 (PMC12425559; doi:10.1111/1759-7714.70163)
Supplement: Supplementary file 1 — Data S1: Supporting Information. [file TCA-16-e70163-s001.docx]

**Supplementary materials and methods**

**Patients and inclusion criteria**

Patients were referred to the host institution for lung resection surgery from eight major Hungarian pulmonary centers. Only patients with primary lung cancer eligible for curative-intent lobectomy were included in the current study. Of note, due to their possible impact on survival, patients who underwent segmentectomy and pulmonectomy were excluded, as well as the patients where the exact date of lung cancer diagnosis was not evident. Importantly, individuals who received neoadjuvant treatment prior to surgery and those who had other oncological diseases and/or distant organ metastases were also excluded. Lastly, in order to eliminate the altering effects of outstanding values, we also excluded all patients with a follow-up period greater than 6 months (or >183 days) caused by undetermined pulmonary nodules that required prolonged monitoring.

**Data collection**

As a general requirement, each patient enrolled in the current study had to undergo either 1) abdominal ultrasound, head CT/MRI and bone scintigraphy, or 2) PET/CT and head CT/MRI. Pathological verification of the lung nodule prior to surgery was not mandatory in every examining center.

The starting date of treatment was defined as the date of the anatomical resection of the primary lung lesion. Surgery was performed by video-assisted thoracic surgery (VATS) and conventional thoracotomy (67.95% and 32.05% of the cases, respectively). Overall survival (OS) was defined as the elapsed time between surgery and death of any cause or last available follow-up. Clinical follow-up was closed on the 1^st^ of January, 2022.

For certain statistical analyses, the elapsed time until surgery was dichotomized. The optimal cut-off value was selected based on its level of significance in defining two distinct subgroups. At first, 30, 60, 77 and 91.06 days between the CT-based diagnosis and surgery were evaluated as possible cut-off values for worse outcomes. Since the most evident differences in outcomes were seen when a using 60 days as cut-off (p=0.002), the subsequent analyses were performed by using this threshold value solely. Accordingly, two patient subgroups were defined for further analyses. Group A consisted of patients who underwent lung resection within 60 days of first suspicion, whereas patients where the time-to-surgery was ≥60 days were categorized as Group B individuals. The inclusion and exclusion criteria along with the patient selection workflow are summarized in Figure 1.

**Survival analysis**

In addition, several additional factors that might have contributed to longer work-up before surgery were as well examined: PET-CT (whether PET-CT was performed and the waiting time before PET-CT), pathological verification, the rank of the examining pulmonary center, and the different phases and waves of COVID-19 pandemic. To investigate the consequences of COVID-19 on the healthcare system more precisely, we defined three different groups with respect to the pandemic waves. The first group included patients diagnosed before the appearance of COVID-19 in Hungary (04.03.2020). The second group included the patients diagnosed during the COVID-19 era, but in time periods when there was no lockdown in the country. We selected patients in the third group if they were diagnosed directly during the COVID waves, thus during the general restrictions in the country. Of note, these pandemic waves lasted from 01.04.2020 to 01.06.2020, from 01.09.2020 to 01.06.2021, and from 01.11.2021 to 31.12.2021 (end of study period).

**Statistical analysis**

Categorical and continuous parameters of the patient cohorts were analyzed by the Chi-square test and by the Student’s t-test. Data distribution was verified by the Kolmogorov-Smirnov normality test. Kaplan-Meier survival curves and two-sided log-rank tests were used for univariate survival analyses. The independent prognostic value of the clinicopathological variables was studied with Cox proportional hazard regression model calculating the hazard ratios (HR) and corresponding 95% confidence intervals (CI). The effect on survival of grouping variables were analyzed by Kruskal-Wallis test and Mann-Whitney U test. P values were always considered statistically significant below 0.05.
